# Methods to reduce the number of vaginal examinations in labour progress assessment so as to support normality at childbirth

**DOI:** 10.1007/s00404-023-07213-3

**Published:** 2023-09-27

**Authors:** Dimitrios Papoutsis, Angeliki Antonakou, Michael Kourakos

**Affiliations:** 1 Midwifery Department, School of Health Sciences, University of Western Macedonia, Kozani, Greece; 2 Midwifery Department, School of Health Sciences, International Hellenic University, Thermi, Greece; 3 Nursing Department, School of Health Sciences, University of Ioannina, Ioannina, Greece

An intervention that has become so routine in labour care that is no longer considered as an intervention is performing vaginal examinations to assess the dilatation of the cervix and the descent of the fetal head in the maternal pelvis. At present, it is considered the gold standard to evaluate labour progression [1]. Establishing a normal or abnormal labour progress is critical, since approximately half of the cases of caesarean section are performed due to abnormal labour progression [2]. The Royal College of Midwives in the United Kingdom has suggested that vaginal examinations be carried out by the same midwife throughout labour so as to reduce inaccuracy and inter-observer variability [3]. The World Health Organization (WHO) and the National Institute for Health and Care Excellence have recommended that vaginal examinations should be performed every 4 h at first stage of labour [4, 5]. WHO has recommended that the number of vaginal examinations should be limited to those which are strictly necessary and ideally should be restricted to the first examination establishing the active phase of labour [6].

Over the years, it has become a matter of debate whether a vaginal examination is the most appropriate method to evaluate labour progression, since it is an invasive procedure associated with pain, embarrassment, potential infection, and possible chorioamnionitis [7]. Moreover, there is inconsistency in examinations with a report that two different clinicians differed in cervical dilatation measurements by 2 cm or more in 11% of occasions [1]. The accuracy also seems to reduce with the increase in cervical dilatation as we approach the second stage of labour [8]. Another study showed that the mean number of vaginal examinations was three when the average labour duration was 8 h, but most importantly, the maximum number of examinations was 11 in some cases [9]. For these reasons and in conjunction with the current interest worldwide to support normality at childbirth, there is an attempt in the literature to identify and re-introduce in clinical practice alternative and less intrusive means of labour progress assessment [6, 7].

In many countries, there is currently a restructuring of maternity services underway with regards to who delivers maternity care, how maternity care is organised, and where maternity care is delivered. The midwifery model of care, which is the maternity model of care provided by midwives and not obstetricians, is considered the ideal and amongst the most effective strategies in supporting normal childbirth [10]. However, the restructuring of maternity healthcare may ultimately not be enough by itself to support normality at birth. The healthcare providers need to re-develop their skills and confidence in non-invasive methods to monitor labour progress such as what existed prior to the significant medicalisation of childbirth in the 1980s [6]. The American College of Obstetricians and Gynecologists quotes that all maternity care providers should be familiar with and should consider using low-interventional approaches and family-centric interventions, when appropriate, for the intrapartum management and assessment of low-risk women in spontaneous labour [11].

There are several methods proposed in the literature either as an alternative or as an adjunct to vaginal examination. These include assessing the frequency and intensity of uterine contractions as well as fetal head descent by abdominal palpation, and identifying changes in maternal behaviour and vocalisations especially in the second stage of labour [7]. As labour advances, it has been suggested that women focus inwards to cope with the intensity of the uterine contractions, vocal changes may be noticed, and the women may appear flushed [12]. At this time point, maternal confidence in preparation for birth needs to be supported, while any unnecessary vaginal examinations may potentially disturb the birthing process and cause undue stress to the woman [12].

Another adjunct tool that has been increasingly researched for the determination of cervical dilatation, fetal head position, and station is the use of intrapartum ultrasound. The World Association of Perinatal Medicine in 2022 issued practice guidelines and recommendations for the use of ultrasound in labour [13]. These report that the use of intrapartum ultrasound is easy, simple, and non-invasive and can serve as an adjunct to correlate alongside findings of a digital vaginal examination. A systematic review in 2022 reported that the sonographic assessment of various fetopelvic parameters to predict labour progress has been proposed as the new gold standard, but more research work needs to be done before this is established [7].

Other non-invasive means of assessing labour progression involve measuring the purple line, that visually appears in 48.3–89.5% of women depending on their ethnicity and skin colour tone [14, 15]. The purple line is the purple discolouration observed from the edge of the anus and extends up to the top of the buttocks as labour progresses and it is absent before active labour [6]. A systematic review in 2022 reported that the purple line length to monitor labour progress had an accuracy of 81–85%, which means that it could potentially identify over 80% of women with normal labour progress [7]. Another recent area of interest involves measuring the transverse diagonal of the Michaelis sacral area which is the area of bone that moves backward when a woman is in advanced labour thus pushing out the wings of the ilea [7].

We believe that the above-mentioned methods do remain promising in the attempt to decrease the number of vaginal examinations during labour, despite the fact that the certainty of evidence for these methods remains ‘low’ to ‘very-low’ [7]. The literature suggests that they can be used alongside vaginal examinations to help establish a correct labour progress assessment. There is no recommended practical guide published yet as to when not to perform a vaginal examination. However, we could avoid a vaginal examination at the start of labour when the purple line is absent, since it only appears in active labour [6]. Moreover, a clinician could potentially reduce the number of vaginal examinations to determine full dilatation and be alternatively guided by changes in maternal vocalisations [12], or when abdominal palpation [7] or intrapartum ultrasound shows significant fetal head descent [13].
